# Pharmacological and non-pharmacological treatment of pediatric primary headaches

**DOI:** 10.1186/1824-7288-40-S1-A84

**Published:** 2014-08-11

**Authors:** Massimiliano Valeriani

**Affiliations:** 1 Headache Center, Neurology, Ospedale Pediatrico Bambino Gesù, IRCCS, Rome, Italy; 2 Center for Sensory-Motor Interaction, Aalborg University, Aalborg, Denmark

## 

Primary headaches represent highly prevalent diseases in childhood and adolescence. Unlike in adulthood, in children the most common primary headache is migraine, while tension-type headache (TTH) is less diffused, especially up to 10 years of age. Cluster headache is to be considered exceptional, showing a prevalence of 0.1%.

While in TTH painful attacks are often of weak intensity and do not require particular treatment, migraine attacks often impair child’s quality of life and need to be interrupted. The first choice drug for the migrainous attack treatment should be ibuprofen. In case of failure, paracetamol and other NSAIDs should be considered. Triptans, which are the most effective drugs for the migraine attack treatment in adults, are not authorized in children, with the exclusion of sumatriptan 10 mg spray.

Whether migraine attacks become too frequent or they do not respond to symptomatic treatment, a prophylactic therapy should be considered. Among drugs used for migraine prophylaxis, only flunarizine and topiramate have solid evidence of effectiveness, although there are also some data in favor of valproate and amitriptyline. It should be underlined that the practice to use minerals or herbs for migraine prophylaxis does not have any scientific support.

Non-pharmacological treatments have also been proposed for pediatric migraine. Cognitive treatment, associated with amitriptyline, has proved useful in chronic migraine. In different painful syndrome, acupuncture was demonstrated to have an analgesic effect. Although a specific demonstration of acupuncture efficacy in pediatric migraine is lacking, this non-pharmacological intervention should be considered, especially in drug resistant cases.

